# A multicentre point prevalence survey of patterns and quality of antibiotic prescribing in Indonesian hospitals

**DOI:** 10.1093/jacamr/dlab047

**Published:** 2021-04-26

**Authors:** Ralalicia Limato, Erni J Nelwan, Manzilina Mudia, Justin de Brabander, Helio Guterres, Enty Enty, Ifael Y Mauleti, Maria Mayasari, Iman Firmansyah, May Hizrani, Raph L Hamers

**Affiliations:** Eijkman-Oxford Clinical Research Unit, Jakarta, Indonesia; Centre for Tropical Medicine and Global Health, Nuffield Department of Medicine, University of Oxford, Oxford, UK; Department of Internal Medicine, Division of Infectious Diseases, Cipto Mangunkusumo National General Hospital, Jakarta, Indonesia; Faculty of Medicine, University of Indonesia, Jakarta, Indonesia; Infectious Disease and Immunology Research Cluster, Indonesian Medical Education and Research Institute, Jakarta, Indonesia; Metropolitan Medical Centre Hospital, Jakarta, Indonesia; Eijkman-Oxford Clinical Research Unit, Jakarta, Indonesia; Eijkman-Oxford Clinical Research Unit, Jakarta, Indonesia; Department of Internal Medicine, Division of Infectious Diseases, Cipto Mangunkusumo National General Hospital, Jakarta, Indonesia; Faculty of Medicine, University of Indonesia, Jakarta, Indonesia; Royal Taruma Hospital, Jakarta, Indonesia; Faculty of Medicine and Health Sciences, Atma Jaya Catholic University, Jakarta, Indonesia; Fatmawati General Hospital, Jakarta, Indonesia; St. Carolus Hospital, Jakarta, Indonesia; Prof. Dr. Sulianti Saroso Infectious Disease Hospital, Jakarta, Indonesia; Metropolitan Medical Centre Hospital, Jakarta, Indonesia; Eijkman-Oxford Clinical Research Unit, Jakarta, Indonesia; Centre for Tropical Medicine and Global Health, Nuffield Department of Medicine, University of Oxford, Oxford, UK; Faculty of Medicine, University of Indonesia, Jakarta, Indonesia

## Abstract

**Background:**

The global emergence of antimicrobial resistance is driven by antibiotic misuse and overuse. However, systematic data in Indonesian hospitals to adequately inform policy are scarce.

**Objectives:**

To evaluate patterns and quality indicators of antibiotic prescribing in six general hospitals in Jakarta, Indonesia.

**Methods:**

We conducted a hospital-wide point prevalence survey (PPS) between March and August 2019, using Global-PPS and WHO-PPS protocols. The analysis focused on antibacterials (antibiotics) for systemic use.

**Results:**

Of 1602 inpatients, 993 (62.0%) received ≥1 antimicrobial. Of 1666 antimicrobial prescriptions, 1273 (76.4%) were antibiotics. Indications comprised community-acquired infections (42.6%), surgical prophylaxis (22.6%), hospital-acquired infections (18.5%), medical prophylaxis (9.6%), unknown (4.6%) and other (2.1%). The most common reasons for antibiotic prescribing were pneumonia (27.7%), skin and soft tissue infections (8.3%), and gastrointestinal prophylaxis (7.9%). The most prescribed antibiotic classes were third-generation cephalosporins (44.3%), fluoroquinolones (13.5%), carbapenems (7.4%), and penicillins with β-lactamase inhibitor (6.8%). According to the WHO AWaRe classification, Watch antibiotics accounted for 67.4%, followed by 28.0% Access and 2.4% Reserve. Hospital antibiotic guidelines were not available for 28.1% of prescriptions, and, where available, guideline compliance was 52.2%. Reason for the antibiotic prescription, stop/review date and planned duration were poorly documented. Culture-guided prescriptions comprised 8.1% of community-acquired infections and 26.8% of hospital-acquired infections.

**Conclusions:**

Our data indicate a high rate of empirical use of broad-spectrum antibiotics in Indonesian hospitals, coupled with poor documentation and guideline adherence. The findings suggest important areas for antimicrobial stewardship interventions.

## Introduction

Drug-resistant infections have been estimated to account for 700 000 deaths per year globally, cumulating to 10 million by 2050, higher than cancer (8.2 million) and diabetes (1.5 million) combined.^1^ The overuse and misuse of antimicrobial agents has been well recognized as one of the key drivers of emerging antimicrobial resistance (AMR),^2^^,^^3^ with antimicrobial consumption projected to rise further globally.^4^ In response to the emerging public health crisis of AMR, the WHO has launched a global action plan, including strategies for surveillance and mitigation of antimicrobial overuse.^5^

Indonesia, a populous (271 million) and diverse middle-income country, is potentially an AMR hotspot, due to persistently high infectious disease burdens, including respiratory infections, diarrhoeal diseases and TB among others,^6^ coupled with liberal antibiotic practices and fragile health systems.^7^^,^^8^ The Indonesian government is increasingly supporting antimicrobial stewardship (AMS), through the national action plan on AMR launched in 2014,^9^^,^^10^ and as part of hospital accreditation.^11^ National antibiotic guidelines were released in 2011,^12^ but have not been updated since. However, inappropriate or unnecessary antibiotic prescribing is believed to be widespread, although systematic data are lacking to adequately inform AMS policies.

In global datasets reporting point prevalence surveys (PPS) of antibiotic use in hospitals,^13^^,^^14^ low and middle-income countries (LMICs) remain underrepresented.^15^^,^^16^ The recently introduced WHO AWaRe (Access, Watch, Reserve) antibiotic classification framework, based on accessibility versus AMR potential, is a useful metric to provide an indication of the appropriateness of antibiotic consumption.^17^^,^^18^

We performed a hospital-wide PPS across six acute-care, general hospitals in Jakarta, the capital city of Indonesia, with the aim of evaluating patterns and quality indicators of antibiotic prescribing. We assessed community and hospital-acquired infections as well as medical and surgical prophylaxis, by hospital, ward type and diagnosis.

## Patients and methods

### Study design and population

We conducted a hospital-wide PPS of antimicrobial use in a purposive sample of six hospitals across Jakarta, between March and August 2019. We followed Global PPS (2018)^19^ and WHO (2019)^13^ protocols. Briefly, a PPS is a ‘snapshot’ survey to collate medical record data on antimicrobial prescriptions in hospitalized patients. Eligible patients were all hospitalized patients who received ≥1 active (i.e. currently ongoing) antimicrobial by 8 a.m. on the survey day or surgical prophylaxis ≤24 h prior to the survey. We excluded emergency and day-care wards, outpatient clinics and inpatients who were discharged before or admitted after 8 a.m.

### Ethical considerations

The study was approved by the Research Ethics Committee of the Faculty of Medicine of the University of Indonesia (1364/UN2.F1/ETIK/2018) and the Oxford Tropical Research Ethics Committee (559-18). The requirement for individual patient consent was waived. Permission was obtained from the hospital management or research/medical committee in each participating hospital.

### Data collection

We developed a paper data collection form (DCF) comprising ward, patient and treatment sections, modified from Global-PPS^19^ and WHO-PPS (Appendix S1, available as Supplementary data at *JAC-AMR* Online).^13^ Data collection was conducted by one or two medical doctors from the study team, joined by one to four junior hospital doctors, who received 1 day of training and a DCF completion guideline. Each ward was completely surveyed within 1 day (to minimize the effect of patient movements) and all wards of a single hospital were surveyed within 4 weeks. De-identified data were extracted from medical notes, drug chart and/or laboratory records; if crucial data were missing or unclear (e.g. unclear writing, mismatch between diagnosis and antimicrobial treatment, missing culture result), the responsible ward nurse or clinician was asked for clarifications. The completed DCFs were entered into a study database. The data coding was verified at two stages: (i) queries during DCF completion were directly resolved with a senior team member (R.L., R.L.H.); and (ii) database inconsistencies were checked against the source data in the medical records as needed.

We included systemic antimicrobials coded on the basis of the WHO Anatomical Therapeutic Chemical (ATC) classification system as follows: antibacterials (J01), antimycotics (J02), antifungals (D01BA), antimycobacterials (J04), antivirals (J05), nitroimidazole derivatives (P01AB), intestinal anti-infectives (A07A) and antimalarials (P01B). We recorded the diagnosis/reason for the prescribed antimicrobial (what the clinician aimed at treating or preventing), according to a diagnostic code list^19^ (Table S1). Antimicrobial indications were classified as: (i) community-acquired infection (CAI) if symptoms were present on admission or started <48 h after admission; (ii) hospital-acquired infection (HAI) if symptoms started ≥48 h after admission; (iii) medical prophylaxis; (iv) surgical prophylaxis, categorized as single-dose, 1 day or longer than 1 day; (v) other; and (vi) unknown. We recorded the following five quality indicators of prescribing: (i) documentation of diagnosis/reason for antimicrobial use, stop/review date and treatment duration in the patient records; (ii) hospital antibiotic guideline availability (i.e. based on a review of all local guidelines by the study team) and compliance with regards to drug choice; if not available, this item was recorded as ‘not assessable’; (iii) parenteral administration; (iv) culture sample taken in therapeutic use; and (v) targeted (antibiotic prescribed in response to microbiology results) or empirical treatment.

### Statistical analysis

We used descriptive statistics to summarize the data, expressed as counts or percentages, by hospital, ward type, indication and diagnosis. The analysis focused on antibacterials (antibiotics) for systemic use (ATC code J01). Antibiotics were reported by drug names, chemical class (according to the fourth level WHO ATC classification) and AWaRe groups. We used RStudio Version 1.3.1093 for all analyses.

## Results

### Hospital characteristics

The six participating hospitals varied by care level (four secondary, two tertiary); sector (three private, three public); availability of hospital antibiotic guidelines (five yes, one no); inclusion in the national health insurance scheme (four yes, two no). All hospitals had an antibiotic stewardship team. All 238 inpatient wards surveyed included 87 medical, 31 surgical, 95 mixed medical-surgical wards and 25 ICUs, of which there were 123 adult, 51 paediatric-neonatal and 64 mixed adult-paediatric-neonatal wards (Table 1). On the survey day, a total of 2358 active hospital beds (median 230, range 134–853 per hospital) accounted for 1602 (67.9%) admitted patients (median 149, range 51–625 per hospital), of whom 993 (62.0%) received ≥1 antimicrobial (median 91, range 33–368 per hospital). Across the hospitals, 53.5% to 78.8% of patients received ≥1 antimicrobial (Table S2).

**Table 1.** Hospital characteristics

	Total	Hospital 1	Hospital 2	Hospital 3	Hospital 4	Hospital 5	Hospital 6
Level of health service	—	secondary	tertiary	secondary	secondary	tertiary	secondary
Sector	—	private	public	private	public	public	private
Teaching hospital	—	yes	yes	yes	no	yes	no
National health insurance scheme^a^	—	no	yes	yes	yes	yes	no
Hospital antibiotic guidelines	—	no	yes	yes	yes	yes	yes
Inpatient wards^b^	238	19	74	30	14	79	22
medical wards	87	4	27	15	6	30	5
surgical wards	31	0	17	2	0	11	1
mixed medical-surgical wards^c^	95	12	19	10	7	32	15
ICUs	25	3	11	3	1	6	1
adult	123	8	55	13	2	39	6
paediatric and/or neonatal	51	5	12	6	3	21	4
mixed adult-neonatal-paediatric^d^	64	6	7	11	9	19	12
Inpatient beds	2358	159	767	300	145	853	134
Admitted patients	1602 (67.9)	100 (62.9)	562 (73.3)	198 (66.0)	66 (45.5)	625 (73.3)	51 (38.1)
Admitted patients on ≥1 antimicrobial	993 (62.0)	75 (75.0)	368 (65.5)	106 (53.5)	52 (78.8)	359 (57.4)	33 (64.7)
medical ward	310 (31.2)	47 (62.7)	102 (27.7)	45 (42.5)	14 (26.9)	95 (26.5)	7 (21.2)
surgical ward	184 (18.5)	0 (0.0)	87 (23.6)	24 (22.6)	0 (0.0)	73 (20.3)	0 (0.0)
mixed medical-surgical ward^c^	401 (40.4)	23 (30.7)	135 (36.7)	37 (34.9)	35 (67.3)	147 (40.9)	24 (72.7)
ICU	98 (9.9)	5 (6.7)	44 (12.0)	0 (0.0)	3 (5.8)	44 (12.3)	2 (6.1)
adult ward	727 (73.2)	31 (41.3)	298 (81.0)	92 (86.8)	43 (82.7)	230 (64.0)	33 (100)
paediatric ward	163 (16.4)	13 (17.4)	58 (15.7)	8 (7.5)	3 (5.7)	81 (22.6)	0 (0.0)
mixed adult-neonatal-paediatric ward^d^	103 (10.4)	31 (41.3)	12 (3.3)	6 (5.7)	6 (11.6)	48 (13.4)	0 (0.0)

Data shown reflect the hospital situation on the survey day, and are expressed as number (percentage), unless otherwise specified.

a National health insurance scheme, Jaminan Kesehatan Nasional (JKN).

b Includes all inpatient wards in the hospital. Some wards have been further subdivided for the purpose of this survey.

c Wards that can admit both medical and surgical patients.

d Wards that can admit adult, paediatric and neonatal patients.

### Patient characteristics

Of 993 patients, 497 (50.1%) were women and 782 (78.8%) were adults, and the median age was 43 years (IQR 22–58.5; range 1 day to 99 years) (Table 2). One or more comorbidities were documented in 48.9% (486) of patients. 299 (30.1%) had been hospitalized in the last 90 days, and 145 (14.6%) had been transferred from another hospital. The 993 patients receiving ≥1 antimicrobial accounted for a total of 1666 active antimicrobial prescriptions (median 1 per patient, range 1–12), with 60.6% (602) receiving one antimicrobial agent, 25.6% (254) two, and 13.8% (137) three or more. Antimicrobial use was highest in ICUs (86.8%, 132/152), followed by surgical wards (66.0%, 184/293), mixed medical-surgical wards (65.0%, 401/622) and medical wards (51.4%, 310/569). Concomitant use of ≥2 antimicrobials was more frequent in ICUs (59.1%, 58/98) than non-ICUs (37.2%, 333/895) (Table S2).

**Table 2.** Characteristics of patients receiving ≥1 antimicrobial

	Total (*n *=* *993)	Hospital 1 (*n *=* *75)	Hospital 2 (*n *=* *368)	Hospital 3 (*n *=* *106)	Hospital 4 (*n *=* *52)	Hospital 5 (*n *=* *359)	Hospital 6 (*n *=* *33)
Female	497 (50.1)	41 (54.7)	186 (50.5)	61 (57.5)	22 (42.3)	175 (48.7)	12 (36.4)
Age, median (IQR)^a^	43 (22–58.5)	29 (20–55)	47 (25–60)	52 (33–66)	39.5 (28–59)	37 (8–53)	51 (28.5–65)
	45 (4.5)	2 (2.7)	17 (4.6)	2 (1.9)	1 (1.9)	23 (6.4)	0 (0.0)
1–23 months	63 (6.3)	3 (4.0)	20 (5.4)	2 (1.9)	1 (1.9)	37 (10.3)	0 (0.0)
2–17 years	103 (10.4)	12 (16.0)	24 (6.5)	6 (5.7)	4 (7.7)	57 (15.9)	0 (0.0)
18–29 years	131 (13.2)	21 (28.0)	47 (12.8)	7 (6.6)	8 (15.4)	39 (10.9)	9 (27.3)
30–39 years	112 (11.3)	9 (12.0)	35 (9.5)	24 (22.6)	12 (23.1)	30 (8.4)	2 (6.1)
40–49 years	145 (14.6)	7 (9.3)	55 (14.9)	7 (6.6)	8 (15.4)	63 (17.5)	5 (15.2)
≥50 years	394 (39.7)	21 (28.0)	170 (46.2)	58 (54.7)	18 (34.6)	110 (30.6)	17 (51.5)
National health insurance holder^b^	743 (74.8)	0 (0.0)	329 (89.4)	24 (22.6)	50 (96.2)	340 (94.7)	0 (0.0)
Transfer from other hospital	145 (14.6)	2 (2.7)	70 (19.0)	2 (1.9)	10 (19.2)	57 (15.9)	4 (12.1)
Hospitalization within 90 days^c^	299 (30.1)	12 (16.0)	86 (23.4)	20 (18.9)	20 (38.5)	151 (42.1)	10 (30.3)
Surgery in the past 90 days^d^	368 (37.1)	3 (4.0)	162 (44.0)	41 (38.7)	8 (15.4)	142 (39.6)	12 (36.4)
Catheter use							
central vascular	132 (13.3)	4 (5.3)	35 (9.5)	12 (11.3)	1 (1.9)	73 (20.3)	7 (21.2)
peripheral vascular	941 (94.8)	69 (92.0)	357 (97.0)	85 (80.2)	51 (98.1)	347 (96.7)	32 (97.0)
urinary	363 (36.6)	9 (12.0)	183 (49.7)	35 (33.0)	9 (17.3)	119 (33.1)	8 (24.2)
intubation	65 (6.5)	3 (4.0)	21 (5.7)	7 (6.6)	1 (1.9)	30 (8.4)	3 (9.1)
Documented comorbidity	486 (48.9)	11 (14.6)	209 (56.8)	40 (37.7)	41 (78.8)	174 (48.5)	14 (42.4)
malnutrition	335 (33.7)	0 (0.0)	165 (44.8)	17 (16.0)	28 (53.8)	121 (33.7)	4 (12.1)
diabetes mellitus	161 (16.2)	8 (10.7)	68 (18.5)	24 (22.6)	14 (26.9)	39 (10.9)	8 (24.2)
TB	120 (12.1)	3 (4.0)	37 (10.1)	7 (6.6)	24 (46.2)	44 (12.3)	5 (15.2)
HIV	44 (4.4)	3 (4.0)	10 (2.7)	2 (1.9)	13 (25.0)	16 (4.5)	0 (0.0)
HIV on ART	27 (2.7)	3 (4.0)	4 (1.1)	2 (1.9)	8 (15.4)	10 (2.8)	0 (0.0)
COPD	11 (1.1)	0 (0.0)	7 (1.9)	0 (0.0)	3 (5.8)	1 (0.3)	0 (0.0)
McCabe score^e^							
rapidly fatal	29 (2.9)	3 (4.0)	8 (2.2)	2 (1.9)	0 (0.0)	15 (4.2)	1 (3.0)
ultimately fatal	217 (21.9)	3 (4.0)	89 (24.2)	32 (30.2)	2 (3.8)	84 (23.4)	7 (21.2)
non-fatal	746 (75.1)	69 (92.0)	270 (73.4)	72 (67.9)	50 (96.2)	260 (72.4)	25 (75.8)
unknown	1 (0.1)	0 (0.0)	1 (0.1)	0 (0.0)	0 (0.0)	0 (0.0)	0 (0.0)
Prescribed antimicrobial drugs	1666	114	630	158	98	622	44
median (range) per patient	1 (1–12)	1 (1–12)	1 (1–9)	1 (1–7)	1 (1–6)	1 (1–10)	1 (1–4)
1	602 (60.6)	58 (77.3)	204 (55.4)	74 (69.8)	32 (61.5)	209 (58.2)	25 (75.8)
2	254 (25.6)	11 (14.7)	108 (29.3)	22 (20.8)	8 (15.4)	99 (27.6)	6 (18.2)
≥3	137 (13.8)	6 (8.0)	56 (15.2)	10 (9.4)	12 (23.1)	51 (14.2)	2 (6.1)

Data shown reflect the hospital situation on the survey day, and are expressed as number (percentage), unless otherwise specified.

a Median (IQR) age was 47 (28–60) years for adults, 7 (2–11) months for children <2 years and 8 (3–14) days for neonates.

b Jaminan Kesehatan Nasional (JKN); Unknown for 4 (0.4%) participants.

c Before the current admission, the patient had been hospitalized in the 90 days before the survey date.

d The patient underwent surgery in the past 90 days before the survey date, including surgery prior to and during the current admission.

e McCabe score is a simple subjective method to assess underlying illness severity and classify patients according to a prognosis of rapidly fatal (<1 year), ultimately fatal (1–4 years) and non-fatal (>5 years).^37^

### Antimicrobial agents prescribed

**Figure 1.**
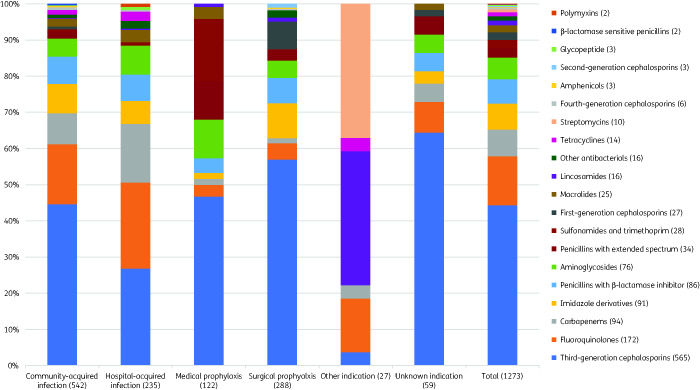
Systemic antibiotic use by antibiotic class, by indication.

Of 1666 antimicrobial prescriptions, 76.4% (1273) were antibiotics (J01), followed by 11.4% (197) antimycobacterials, 4.3% (72) antivirals, 3.7% (62) antimycotics, 2.6% (43) intestinal anti-infectives, 0.8% (13) antimalarials and 0.4% (6) nitroimidazole derivatives. Among 46 different antibiotic agents (J01), the five most prescribed (accounting for 56.5%, 720) were ceftriaxone (26.8%, 341), levofloxacin (10.7%, 137), metronidazole (7.1%, 91), meropenem (6.4%, 82) and cefotaxime (5.6%, 71) (Table S3). The top five antibiotic classes (accounting for 78.0%, 993) were third-generation cephalosporins (44.3%, 565), fluoroquinolones (13.5%, 172), carbapenems (7.4%, 94), penicillins with β-lactamase inhibitor (6.8%, 86) and aminoglycosides (6.0%, 76) (Figure 1).

### Reasons and indications of antibiotic prescriptions

Table S4 summarizes diagnosis/reasons for all 1666 antimicrobial prescriptions. Among all 1273 antibiotic prescriptions (J01), the most common reasons (accounting for 49.4%, 629) were pneumonia (27.7%, 353), skin and soft tissue infections (8.3%, 106), gastrointestinal prophylaxis (7.9%, 101) and gastrointestinal infections (5.4%, 69) (Tables 3 and S5). Ceftriaxone and levofloxacin were mainly prescribed for pneumonia and gastrointestinal infections; metronidazole for skin and soft tissue infections, intra-abdominal infections and gastrointestinal prophylaxis; and meropenem for pneumonia and sepsis.

**Table 3.** Most common diagnosis for systemic antibiotic use

Diagnosis	Total (*n *=* *1273)
Pneumonia or lower respiratory tract infection	353 (27.7)
Skin and soft tissue infection^a^	106 (8.3)
Prophylaxis for gastrointestinal infections^b^	101 (7.9)
Gastrointestinal infection	69 (5.4)
Prophylaxis for bone and joint infection^c^	58 (4.6)
Prophylaxis for obstetrics or gynaecological infection	58 (4.6)
Intra-abdominal infection^d^	57 (4.5)
Unknown reason	47 (3.7)
Sepsis	46 (3.6)
Prophylaxis for urinary tract infection (surgery or recurrent infection)	42 (3.3)
Other diagnosis^e^	35 (2.7)
Ear, nose, throat infection^f^	30 (2.4)
Upper urinary tract infection^g^	29 (2.3)
Central nervous system infection	26 (2.0)
Medical prophylaxis for newborn risk factors	25 (2.0)

The table lists the 15 most common reasons to prescribe at least one antibiotic for systemic use (J01). Data are expressed as number (percentage) and ranked by frequency. Patients recorded with more than one diagnosis were counted by number of diagnoses. Diagnoses were coded based on the GLOBAL-PPS 2018 Diagnostic Code List (Table S1). The full list of diagnosis is shown in Table S5.

a Including cellulitis, wound including surgical site infections, deep soft tissue not involving bone (e.g. infected pressure or diabetic ulcers, abscess).

b Including prophylaxis for surgery of the gastrointestinal tract, liver or biliary tree, and prophylaxis in patients with neutropenia or hepatic failure.

c Including prophylaxis for surgical site infections, for plastic or orthopaedic surgery (bone or joint).

d Including hepatobiliary, intra-abdominal abscess, etc.

e Antibiotic prescribed with documentation for which there is no above diagnosis group.

f Including mouth, sinuses, larynx.

g Including catheter-related urinary tract infection, pyelonephritis.

The most common antibiotic indication was CAI (42.6%, 542), followed by surgical prophylaxis (22.6%, 288), HAI (18.5%, 235), medical prophylaxis (9.6%, 122), unknown (4.6%, 59) and other (2.1%, 27). The top five CAI were pneumonia (42.6%, 231), skin and soft tissue infection (14.2%, 77), gastrointestinal infection (12.2%, 66), sepsis (5.5%, 30), and intra-abdominal infection (5.4%, 29) (Table S5); top five antibiotics were ceftriaxone (32.8%, 178), levofloxacin (13.5%, 73), metronidazole (8.1%, 44), meropenem (7.7%, 42) and ampicillin/sulbactam (5.4%, 29) (Table S6). Hospital-acquired pneumonia (including other HAI) was the most common HAI (70.2%, 165), followed by intervention-related infections (including catheter-related blood stream infection, ventilator-associated pneumonia, catheter-related urinary tract infection) (19%, 35), post-operative surgical site infection (13.6%, 32) and infection present on admission from another hospital (0.85%, 2) or long-term care facility (0.4%, 1); no *Clostridioides difficile*-associated diarrhoea was documented (Table S7). The top five antibiotics for HAI were levofloxacin (18.7%, 44), meropenem (13.6%, 32), ceftriaxone (9.8%, 23), amikacin (6.4%, 15), metronidazole and ceftazidime (6%, 14 each) (Table S6).

The top five reasons for medical prophylaxis were neonatal (20.5%, 25), general (19.7%, 24), gastrointestinal (17.2%, 21), respiratory (15.6%, 19) and unknown (8.2%, 10) (Table S5); the top five antibiotics were ceftriaxone (28.7%, 35), cotrimoxazole (17.2%, 21), gentamicin (10.7%, 13), cefotaxime (8.2%, 10) and ampicillin (7.4%, 9) (Table S6). The top five reasons for surgical prophylaxis were gastrointestinal (27.8%, 80), obstetrics/gynaecology (20.1%, 58), bone and joint (17%, 49), urinary tract (12.8%, 37), central nervous system and ear-nose-throat (7.3%, 21 each); the top five antibiotics were ceftriaxone (26.4%, 76), cefixime (11.5%, 33), cefoperazone (11.1%, 32), metronidazole (9.7%, 28) and cefazolin (6.2%, 18). Notably, the duration of surgical prophylaxis was longer than 1 day for 76% (219) of prescriptions, whereas 15.0% (43) was single-dose and 9.0% (26) was for 1 day.

### AWaRe classification

**Figure 2.**
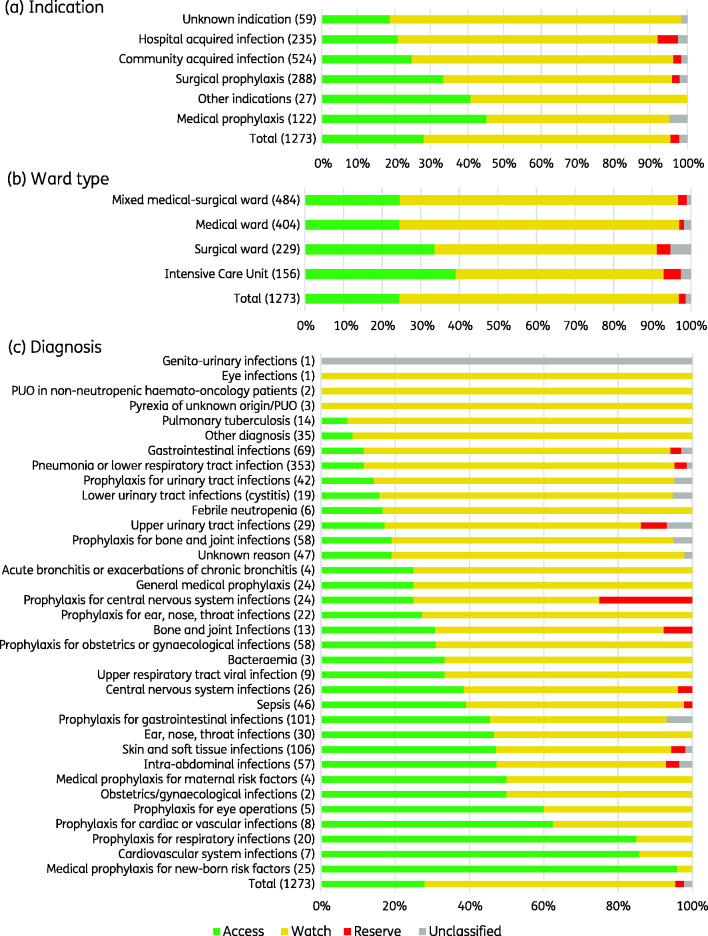
Systemic antibiotic use by AWaRe classification.

**Figure 3.**
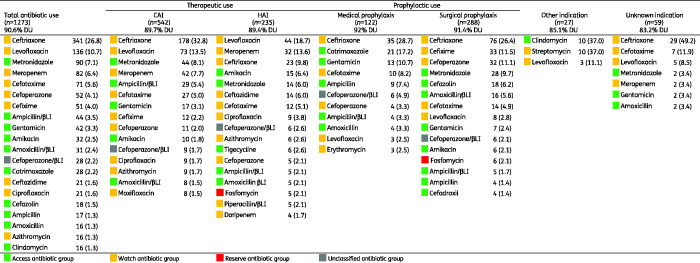
Systemic antibiotic use by indication based on AWaRe classification. Data are expressed as numbers (percentage). Antibacterial prescriptions for systemic use (J01) were included. DU 90%, the number of drugs which account for 90% of the prescriptions; CAI, community-acquired infection; HAI, hospital-acquired infection; βLI, β-lactamase inhibitor.

Of all 1273 antibiotic prescriptions (J01), 67.4% (858) were Watch antibiotics, followed by 28.0% (356) Access, 2.4% (31) Reserve and 2.2% (28) Unclassified (Figure 2). This pattern was similar across indications and ward types. The Watch top five were ceftriaxone (39.7%, 341), levofloxacin (15.8%, 136), meropenem (9.6%, 82), cefotaxime (8.3%, 71) and cefoperazone (6%, 52). Notably, Watch antibiotics were commonly prescribed for the most frequent diagnoses, i.e. pneumonia (34.4%, 295), gastrointestinal infection (6.6%, 57) and skin and soft tissue infection (5.8%, 50). The Access top five were metronidazole (25.3%, 90), ampicillin/sulbactam (12.4%, 44), gentamicin (11.8%, 42), amikacin (9%, 32) and amoxicillin/clavulanic acid (8.7%, 31). Reserve antibiotics were uncommon, and included fosfomycin (15, 48.4%), tigecycline (13, 41.9%), colistin (1, 3.2%) and linezolid (1, 3.2%) (Figure 3, Table S8).

### Quality indicators of antibiotic prescribing

#### Documentation of antibiotic plan

The reason for prescribing was documented for 63.5% (808/1273) of all prescriptions. Documentation of diagnosis/reason was better for therapeutic use (546/777, 70.3%) than prophylactic use (230/410, 56.0%), and in ICUs (75.6%, 118/156) than non-ICUs (61.8%, 690/1117). Stop/review date (15.2%, 194/1273) and planned treatment duration (9.8%, 125/1273) were poorly documented overall (Table 4).

**Table 4.** Quality indicators for antibiotic prescribing, by indication

Quality indicators	Total (*n *=* *1273)	Therapeutic use (*n *=* *777)					Prophylactic use (*n *=* *410)		Other indication^a^ (*n *=* *27)	Unknown indication (*n *=* *59)
		CAI empirical (*n *=* *498)	CAI targeted (*n *=* *44)	HAI empirical (*n *=* *172)	HAI targeted (*n *=* *63)		Medical prophylaxis (*n *=* *122)	Surgical prophylaxis (*n *=* *288)		
Reason documented	808 (63.5)	321 (64.5)	35 (79.5)	145 (84.3)	45 (71.4)		58 (47.5)	172 (59.7)	21 (77.8)	11 (18.6)
Stop/review date documented	194 (15.2)	69 (13.9)	7 (15.9)	43 (25.0)	18 (28.6)		15 (12.3)	38 (13.2)	2 (7.4)	2 (3.4)
Treatment duration documented	125 (9.8)	35 (7.0)	5 (11.4)	31 (18.0)	12 (19.0)		3 (2.5)	35 (12.2)	0 (0.0)	4 (6.8)
Guideline compliance										
yes	478 (37.5)	223 (44.8)	17 (38.6)	79 (45.9)	33 (52.4)		23 (18.9)	82 (28.5)	21 (77.8)	0 (0.0)
no	378 (29.7)	136 (27.3)	16 (36.4)	70 (40.7)	20 (31.7)		6 (4.9)	124 (43.1)	6 (22.2)	0 (0.0)
not assessable^b^	358 (28.1)	139 (27.9)	11 (25.0)	23 (13.4)	10 (15.9)		93 (76.2)	82 (28.5)	0 (0.0)	0 (0.0)
indication unknown	59 (4.6)	0 (0.0)	0 (0.0)	0 (0.0)	0 (0.0)		0 (0.0)	0 (0.0)	0 (0.0)	59 (100)
Route of administration										
parenteral (IV)	1084 (85.2)	444 (89.2)	42 (95.5)	150 (87.2)	58 (92.1)		92 (75.4)	237 (82.3)	10 (37.0)	51 (86.4)
oral	183 (14.4)	52 (10.4)	2 (4.6)	22 (12.8)	5 (7.9)		30 (24.6)	51 (17.7)	13 (48.2)	8 (13.6)
IV to oral switch	48 (26.2)	6 (11.5)	1 (50.0)	9 (40.9)	1 (20.0)		1 (3.3)	28 (54.9)	0 (0.0)	2 (25.0)
other	6 (0.47)	2 (0.40)	0 (0.00)	0 (0.00)	0 (0.00)		0 (0.00)	0 (0.00)	4 (14.81)	0 (0.00)
Culture sample taken^c^	344 (44.3)	125 (25.1)	44 (100)	112 (65.1)	63 (100)		—	—	—	—

CAI, community-acquired infection; HAI, hospital-acquired infection.

a Other indication included antibiotics prescribed for neurotoxoplasmosis, pulmonary TB and as a motility agent.

b Hospital antibiotic guidelines were not available to assess compliance.

c Only applicable to therapeutic use.

#### Hospital guideline availability and compliance

Local antibiotic guidelines were not available for 28.1% (358/1273) of prescriptions; notably, including 76.2% (93/122) of prescriptions for medical prophylaxis. Guideline compliance for drug choice was 52.2% (478/915) overall, 44.8% (223/498) for empirical CAI treatment, 45.9% (79/172) for empirical HAI treatment, 28.5% (82/288) for surgical prophylaxis and 18.9% (23/122) for medical prophylaxis (Table 4).

#### Parenteral use

In total, 85.1% (1084/1273) of prescriptions were parenterally administered, including 88.5% (208/235) for HAI, 89.5%% (486/542) for CAI, 75.4% (92/122) for medical prophylaxis and 82.3% (237/288) for surgical prophylaxis (Table 4).

#### Culture samples taken

Among 619 patients with ≥1 antibiotic for therapeutic use, 48.8% (302) had ≥1 sample taken for bacterial culture (total 831 samples, median 2, range 1–25 per patient). Blood cultures were taken in 44.4% (88/353) of pneumonias, 45.6% (26/57) of intra-abdominal infections, 58.6% (17/29) of upper urinary tract infections and 95.8% (23/24) of sepsis. Sputum cultures were taken in 26.9% (95/353) of pneumonias. Urine cultures were taken in 72.4% (21/29) of upper urinary tract infections (Table S9).

#### Targeted antibiotic treatment

Treatment was targeted in 8.1% (44/542) of CAI and 26.8% (63/235) of HAI; 13.0% (46/353) of pneumonias, 15.8% (9/57) of intra-abdominal infections, 44.8% (13/29) of upper urinary tract infections and 13.0% (6/46) of sepsis.

## Discussion

This was the first contemporary hospital-wide survey in Indonesia that systematically evaluated patterns and quality of antibiotic prescribing, using the recommended PPS methodology.^13^^,^^19^ We demonstrated the feasibility of PPS in this low-resource setting, and generated useful data to guide local AMS interventions. We found proportions of inpatients in Indonesian hospitals receiving antibiotics to be substantially higher (62%) than reported in global PPS datasets (27%–39%), which were dominated by data from high-income countries in Europe, North America and Asia.^15^^,^^16^ In our survey, antibiotic use varied between hospitals (53%–79% of patients), and was highest in ICUs (86.8%).

Consistent with other surveys in Asia^20^^,^^21^ and globally,^15^ lower respiratory tract infections were the predominant reason for antibiotic prescribing in Jakarta hospitals. In our survey, the most-used antibiotic classes were third-generation cephalosporins (mainly ceftriaxone), fluoroquinolones (mainly levofloxacin) and carbapenems (mainly meropenem), all predominantly used for pneumonia, among several other diagnoses. Ceftriaxone was the most-used antibiotic across all major indications (i.e. CAI, HAI, surgical and medical prophylaxis). These findings are consistent with the widespread use of broad-spectrum antibiotics, predominantly third-generation cephalosporins and fluoroquinolones, in Indonesia,^22^ other Asian countries^20^^,^^21^^,^^23–25^ and globally,^15^^,^^16^ which may suggest that at least a proportion of these prescriptions are unnecessary or inappropriate. Moreover, empirical use of meropenem for CAI and HAI represented nearly 10% of all antibiotics for therapeutic use; this was similar to a globally reported rate of 12.2%,^15^ but substantially higher than the overall 4.1% reported in European countries.^26^ Substantial use of carbapenems in our survey could partially be explained by the fact that two of the six hospitals were tertiary referral centres attending to complex patients, as well as high reported rates of AMR in Indonesian hospitals, particularly in common Gram-negative organisms.^27^ Nonetheless, culture-guided prescribing for CAI (8%) and HAI (27%) was low in comparison to a global study (12%–27% and 20%–44%, respectively)^15^, suggesting underutilization of microbiological diagnostics as well as overuse of broad-spectrum antibiotics.

Antibiotic prescriptions for HAI (18.5% of total), predominantly for pneumonia but also intervention-related and post-operative surgical site infections, were comparable to recent surveys in India (19%)^21^ and Thailand (34%),^20^ but considerably higher than in reports from high-income settings, e.g. ECDC survey (6%)^26^ and the GLOBAL-PPS survey (8.4%).^15^ These data confirm the significantly higher burden of HAI in LMICs compared with high-income countries.

A high proportion of antibiotic prescriptions were for surgical (23%) and medical prophylaxis (10%), for a range of indications. Prophylactic prescribing was unusually high for gastrointestinal infections. Prolonged (>1 day) surgical prophylaxis was very common (76%) in our survey, as has also been observed in other countries in Asia (Pakistan 97%,^23^ India 77%,^21^ Thailand 90%^20^) as well as in Europe.^15^^,^^26^ Prolonged antibiotic prophylaxis for more than 24 h for most surgical indications does not prevent development of postoperative infections, compared with <24 h, but increases the risk of AMR and side-effects.^28^ Further research is warranted to explain the reasons for these patterns.

We investigated five basic quality indicators, which could be used to set benchmarks for quality improvement of antibiotic use^29^ and AMS programmes.^30^ Documentation of the reason of prescribing (64%) was lower than reported across studies in Europe, Asia, Africa and America (70%–85%).^15^^,^^31^ Stop or review date was poorly documented (15%) across indications and ward types. Post-prescription review of a prescribed antimicrobial within 48–72 h of the initial order ensures appropriate choice and route of administration and optimal de-escalation (IV to oral switch) practices and prevents unnecessarily long antibiotic courses. The high (85%) proportion of parenteral route of administration, coupled with high rates of empirical therapy and suboptimal use of microbiological cultures, suggests lack of de-escalation protocols in the participating hospitals. Proactive IV to oral switching policies are recognized as a key metric for AMS processes, and can reduce catheter-related complications, healthcare costs and duration of hospital stays.^32^

A systematic review and meta-analysis showed that guideline-adherent empirical therapy was associated with a relative risk reduction for mortality of 35%.^32^ The reason for poor guideline compliance (52%) in our survey is uncertain and probably multifactorial, including local resistance patterns, ineffective guideline dissemination and clinical uncertainty with fear of treatment failure. Our findings should trigger further detailed investigations at hospital and country level.

The WHO AWaRe framework offers an attractive metric for LMICs in the absence of validated quality indicators for antibiotic appropriateness,^17^^,^^33^^,^^34^ and includes a > 60% national target of total antibiotic consumption in the Access category by 2023.^35^ However, a recent assessment of antibiotic consumption data from 76 countries in 2000–15 found that the global per-capita consumption of Watch antibiotics increased by 90.9%, compared with an increase of 26.2% in Access antibiotics, with disproportionate increases in Watch antibiotic consumption in LMICs (165% compared with 27.9% in high-income countries).^18^ Although Indonesia national-level data have not been included in the AWaRe reports to date,^35^ our survey found hospital consumption of Access antibiotics at 28% to be below the 60% target, mostly driven by ceftriaxone and levofloxacin use for CAI and HAI. Although these findings could partially be explained by the national health insurance scheme which determines available antibiotics based on the national formulary,^36^ they also highlight significant challenges for AMS.

Limitations of this study are inherent to the cross-sectional PPS design, providing a mere snapshot of the antibiotic situation in the hospital surveyed. Moreover, given that we used a convenient sample of six hospitals in metropolitan Jakarta, which are potentially better resourced than many other hospitals in Indonesia, data are not necessarily representative for all hospitals in Indonesia, urging caution in extrapolating the observed patterns. Indeed, antibiotic prescribing can be influenced by many factors, e.g. patient case-mix, prevalence of different types of infections, AMR patterns and institutional factors.

In conclusion, we observed high levels of parenteral, empirical use of broad-spectrum antibiotics in Indonesian hospitals, and inadequate performance on key quality indicators of prescribing. Despite important progress in AMS, supported by national policies,^10^^,^^11^ the study findings highlighted the need to strengthen AMS to increase use of narrower-spectrum antibiotics through culture-guided, targeted treatment and hospital guideline compliance. Further research is needed to understand the complex drivers of antibiotic prescribing, and to develop context-specific and feasible quality improvement strategies to strengthen existing AMS programmes.

## Supplementary Material

dlab047_Supplementary_Data

## EXPLAIN study group

Ralalicia Limato, Erni J. Nelwan, Manzilina Mudia, Helio Guterres, Enty Enty, Ifael Y. Mauleti, Maria Mayasari, Iman Firmansyah, May Hizrani, Raph L. Hamers, Anis Karuniawati, Prof Taralan Tambunan, Prof Amin Soebandrio, Prof Henri Verbrugh, Decy Subekti, Iqbal Elyazar, Mutia Rahardjani, Fitria Wulandari, Prof Reinout van Crevel, Rogier van Doorn, Vu Thi Lan Huong, Nga Do Ti Thuy, Sonia Lewycka, Prof Alex Broom.

## Supplementary data

Appendix S1 and Tables S1 to S9 are available as Supplementary data at *JAC-AMR* Online.
